# High-Throughput Sequencing and Metabolomics Reveal Differences in Bacterial Diversity and Metabolites Between Red and White Sufu

**DOI:** 10.3389/fmicb.2020.00758

**Published:** 2020-04-22

**Authors:** Guiliang Tan, Min Hu, Xueyan Li, Ziqiang Pan, Mei Li, Lin Li, Maoxun Yang

**Affiliations:** ^1^School of Material Science and Food Engineering, Zhongshan Institute, University of Electronic Science and Technology of China, Zhongshan, China; ^2^Guangdong Key Laboratory of Integrated Agro-Environmental Pollution Control and Management, Guangdong Institute of Eco-Environmental Science and Technology, Guangdong Academy of Sciences, Guangzhou, China; ^3^National-Regional Joint Engineering Research Center for Soil Pollution Control and Remediation in South China, Guangzhou, China; ^4^Zhuhai Da Hengqin Science and Technology Development Co., Ltd., Zhuhai, China

**Keywords:** red sufu, white sufu, bacterial community analysis, metabolite, high-throughput 16S rRNA gene sequencing

## Abstract

Sufu is a traditional fermented soybean food produced in China. However, the microbial compositions and metabolites of different types of sufu have not been studied in detail. Accordingly, in this study, we evaluated the differences in bacterial communities and metabolites between commercial red sufu (RS) and white sufu (WS). Principal coordinate analysis and the unweighted pair group method with arithmetic means analysis of 16S rRNA genes revealed that the bacterial community structures of RS and WS differed dramatically. At the phylum level, the relative abundances of *Firmicutes* and *Proteobacteria* were significantly different between RS and WS (*P* < 0.01). Moreover, the abundances of *Lactococcus* and *Tetragenococcus* genera were significantly different between RS and WS (*P* < 0.01). Among metabolites, most free amino acids, few of volatile flavor compounds, and some organic acids showed significant differences between RS and WS (*P* < 0.05). Additionally, correlations between microbiota and metabolites were determined. Aggregated boosted tree analysis showed that formic acid had the highest relative influence (20.27%) on bacterial community diversity (Chao 1), following by arginine (5.38%), propanol (4.57%), oxalic acid (4.46%), and hexanol (4.43%). Moreover, *Streptococcaceae* and *Moraxellaceae* had the highest relative influence on the concentration of formic acid (12.84% and 8.75%, respectively). The profiles obtained in this study improve our understanding of the relationships between bacterial flora and metabolites in different types of sufu. These findings may help us interpret the roles of bacterial communities in the flavor and characteristics of sufu.

## Introduction

Sufu (also called “oriental cheese”) is a traditional fermented soybean food in China that shares similar shapes, textures, and fermentation mechanisms with cheese (Xie et al., 2018). Sufu is normally consumed as a flavor enhancer and appetizer owing to its characteristic flavor, pleasant taste, and nutrition value in China and other Asia countries (Han et al., 2001b), with an estimated annual production of over 300,000 tons in China (Xia et al., 2014). Based on the strain used as a starter, sufu can be classified into three different types, i.e., mold-fermented sufu (inoculated with *Actinomucor*, *Mucor*, or *Rhizopus*), bacteria-fermented sufu (inoculated with *Bacillus* or *Micrococcus*), and naturally inoculated sufu (no artificial addition of microorganisms; Feng et al., 2013; Xie et al., 2018). However, based on differences in color and flavor, sufu is mainly categorized as red sufu (RS) or white sufu (WS) in the product market (Han et al., 2004a; Xie et al., 2018). RS contains red colorant (angkak), whereas WS is free of colorant (Ho et al., 1989; Han et al., 2001b). Interestingly, these two types of sufu contain different contents of metabolites, such as free amino acids (FAAs), organic acids, and volatile flavor compounds (VFCs; Han et al., 2001b, 2004b; Xie et al., 2018, 2019), and show differences in lactic acid bacteria (LAB) compositions (Han et al., 2001a). For example, the levels of FAAs were reported to be higher in WS than in RS. Sufu is produced under open or semi-open conditions via the actions of various microbes, including starter and indigenous microorganisms, which play important roles in flavor generation (Han et al., 2001b; Feng et al., 2013; Huang et al., 2018; Xie et al., 2018). Generally, four steps are involved in sufu manufacture: Firstly, soybeans are washed and soaked in water, and then ground into a slurry. The slurry is diluted, pressed and filtrated to obtain soymilk. Then, the filtrated soymilk is coagulated by the addition of salts and pressed to produce tofu. After that, tofu is inoculated with mould cultures (such as *Actinmucor elegans*) to prepare pehtze. Subsequently, salting of pehtze with a saturated salt solution, and finally, ripening of sufu by added of a dressing mixture. The dressing mixture of RS mainly consists of salt, angkak (red kojic rice), alcoholic beverage, sugar, and flour or (soybean) paste and some spices. The WS has similar ingredients as RS in the dressing mixture but without angkak (Supplementary Figure S1). Exploration of the relationships between microbial communities and metabolites is crucial for improvement of the quality of sufu (Huang et al., 2018; Xie et al., 2018).

Recently, high-throughput sequencing has been introduced to investigate the diversities and compositions of microbiota; these studies have expanded our knowledge of the bacterial community structure in sufu (Huang et al., 2018; Xie et al., 2018; Liang et al., 2019; Wan et al., 2019; Xu et al., 2020). Bacteria belonging to the genera of *Lactococcus*, *Tetragenococcus*, *Streptococcus*, *Enterobacter*, *Acinetobacter*, and *Brevibacterium* are the predominant microorganisms in sufu; however, their abundances are mostly correlated with chemical characteristics, such as salinity, ethanol content, and angkak composition (Hwan and Chou, 1999; Han et al., 2001a). Moreover, metabolomic approaches, such as gas chromatography (GC), mass spectrometry (MS), high-performance liquid chromatography (HPLC), and proton nuclear magnetic resonance have been applied to determine the metabolite profiles of sufu (Moy and Chou, 2010; Huang et al., 2018; Xie et al., 2018; Fan et al., 2019). However, few systematic studies have evaluated differences in microbial compositions and metabolites between RS and WS, which are the most commonly consumed types of sufu in China.

Accordingly, in this study, we aimed to investigate the diversity and composition of bacterial communities, profiles of metabolites (e.g., amino acids, organic acids, and VFCs), and correlations between bacteria and metabolites in RS and WS. The results will contribute to the improvement of our understanding of the roles of bacteria in the production of flavor substances in different types of sufu and thus facilitate the isolation and screening of indigenous strains for the production of high-quality sufu.

## Materials and Methods

### Sample Collection

Commercial sufu samples were randomly purchased from different cities in China in November and December 2018 (Supplementary Table S1). All cube samples were collected in 50-mL Corning CentriStar Centrifuge Tubes (Corning CentriStar, NY, United States), immediately transported on ice to the laboratory, and stored at −20°C until DNA extraction and chemical analysis.

### Determination of Chemical Characteristics

For pH analysis, 10 g sufu was mixed with 100 mL distilled water, heated to a boil, cooled, and then centrifuged (9000 × *g*, 10 min). The pH of the supernatant was measured directly with a PB-10 pH meter (Sartorius, Gottingen, Germany). The NaCl content of the samples was determined using the A.O.A.C. Official Method (937.09). The total acidity (TA) and amino acid nitrogen (AAN) contents were analyzed by the titration method using an automatic potentiometric titrator (905-Titrando; Metrohm, Switzerland), as described previously, with some modifications (Chen et al., 2018). Briefly, the supernatants of samples were automatically titrated with 0.01 M NaOH until the final pH of the solution was 8.2, and the amount of titrant was used to determine the acidity of sufu. Then, excess formaldehyde was added to samples for fixing amino acids. The samples were titrated with 0.05 M NaOH until the final pH of the solution was 9.2, and the amount of titrant was used to determine the AAN content of sufu.

Organic acids were analyzed by HPLC, as previously described (Moy and Chou, 2010), with minor modifications. Briefly, an organic acid analysis column (Atlanti T3; 10.0 × 250 mm, 5 μm; Waters Corp., Milford, MA, United States) was used. The chromatographic conditions were as follows: mobile phase, potassium phosphate solution (pH 2.5); flow rate, 1.0 mL/min; detection wavelength, 210 nm; temperature, 30°C; injection volume, 20 μL. Various mixtures of standard solutions containing oxalic acid, tartaric acid, formic acid, pyruvic acid, malic acid, ascorbic acid, lactic acid, acetic acid, citric acid, succinic acid, and propanoic acid (Sigma-Aldrich, St. Louis, MO, United States) were run on the HPLC (model 1200; Agilent Ltd., United States).

Free amino acids in sufu were detected using ultra- HPLC-tandem MS (UPLC-MS/MS; model 1290/6460; Agilent Ltd.). The samples were extracted with distilled water (pH 3.0), and the extracted solutions were purified using hexane. The FAAs were then separated on an ACQUITY UPLC BEH HILIC (2.1 × 100 mm, 1.7 μm; Waters Corp.) using ammonium formate-acetonitrile/ammonium formate-H_2_O (pH 3.0) as the mobile phase and detected by MS/MS under multiple reaction monitoring modes. The MS measurement was conducted using positive (ESI^+^) and negative (ESI^–^) electrospray ionization with an external standard.

VFCs were analyzed as described previously (Xie et al., 2018), with minor modifications. Briefly, sufu samples (3 g) were mixed with 0.5 g NaCl, followed by equilibration with a thermostatic water bath at 55°C for 15 min. Then, the VFCs were extracted with an SPME fiber (PDMS/DVB/CARB; Supelco Co., Bellefonte, PA, United States) at 55°C for 30 min. The oven temperature gradient of GC-MS started at 33°C (2 min), increased at 5°C/min to 70°C, then increased at 10°C/min to 250°C. The settings were as follows: injector temperature of 250°C and run time of 58 min. An Agilent 6890N GC coupled with an Agilent 5975 Mass Selective Detector (Agilent Ltd.) was used for GC-MS analysis. The compounds were identified by comparison with the mass spectral data from the NIST 14 mass spectral database. All analyses described above were carried out in triplicate.

### Enumeration of Bacteria

Sufu (25 g) and 225 mL sterilized 0.85% NaCl solution were placed in a sterilized homogenous bag and homogenized using a stomacher for 2 min, yielding a 10^–1^ dilution. Then, the homogenized sample solution was serially diluted (10-fold) in sterilized 0.85% NaCl solution. The diluted samples were spread on Plate Count Agar (PCA; Beijing Land Bridge Technology Co., Ltd., China) for enumeration of bacteria. The PCA plates were incubated at 37°C in an incubator for 2 day. The numbers of bacteria were calculated as colony-forming units (CFU) per gram sufu.

### Community DNA Extraction and 16S rRNA Gene Amplicon Sequencing

The total genomic DNA from sufu samples (0.5 g) was extracted using a Powersoil DNA Isolation Kit (Qiagen, Hilden, Germany) according to the manufacturer’s instructions. The yield and quality of DNA were analyzed electrophoretically on 1% agarose gels. The DNA samples were stored at -70°C until analysis. Triplicate samples of extracted DNA from the same sample were combined for downstream analysis. The V4-V5 region of 16S rRNA gene was amplified using specific primers (515F, 5′-GTGCCAGCMGCCGCGGTAA-3′; 926R, 5′-CCGTCAATTCMTTTRAGT-3′; Baker et al., 2003) with a 12-base barcode in the 5′-end of the reverse primer used for sample multiplexing. Polymerase chain reaction (PCR) was performed in a total reaction volume of 50 μL containing 0.5 μL of each primer, 1 μL template DNA, 23 μL dd-H_2_O, and 25 μL of 2 × *ExTaq* PCR Master Mix (Takara Biotechnology, Dalian, China). PCR conditions were as follows: 10 min denaturation at 95°C; 28 cycles of 94°C for 30 s, 55°C for 30 s, and 72°C for 30 s; and a final extension at 72°C for 10 min. Three replicate PCR products per sample were pooled and purified using a QIAquick PCR purification kit (Qiagen). The amplicons were subjected to 2% agarose gel electrophoresis, and quantity was assessed using a Qubit 3.0 Fluorometer (ThermoFisher Scientific, United States). The purified amplicons were mixed at equimolar amounts before being sequenced on an Illumina Hiseq2500 platform using 250 bp pair-end reads at Beijing Novogene, Beijing, China^1^.

### Bioinformatics Analysis of 16S rRNA Gene Amplicon Sequences

Using the default settings in QIIME2 (Caporaso et al., 2010), sequences shorter than 200 bp and low-quality reads (average base quality <25) were removed. In addition, sequences containing more than six ambiguous nucleotides (“N”) were removed. The sequences were then assigned to each sample based on their 12-bp barcode. Sequences from all samples were clustered into operational taxonomic units (OTUs) at 97% sequence similarity using UCLUST v6.1 (Edgar et al., 2011), with an open-reference OTU picking strategy by applying the Greengenes 16S rRNA database as a reference (DeSantis et al., 2006). Representative sequences in each OTU were assigned to taxonomic groups using the RDP classifier (Cole et al., 2006) within an 80% confidence threshold. Chimeric OTUs were identified and removed using UCHIME implemented in QIIME2 (Edgar et al., 2011). A phylogenetic tree was generated from the alignment file using FastTree2 (Price et al., 2010). Finally, to estimate alpha diversity, a random subsampling method for each sequence library was used for microbial community diversity index calculations to control for the effects of library size. Alpha diversity indices (Chao1, Goods coverage, phylogenic diversity [PD], Shannon, and Simpson) were calculated for all samples with 1,000 repetitions using a size of 55,230 sequences per sample. For beta diversity analysis, all samples were also subsampled to 55,230 sequences per sample to remove sample-size effects. Principal coordinate analysis (PCoA) plots and unweighted pair group method with arithmetic means analysis (UPGMA) hierarchical clustering were performed using Bray–Curtis distances in the QIIME2 software package.

### Richness, Abundance, and Identity of Taxa Shared Between RS and WS

Core taxa across samples were obtained from the taxa-abundance matrix (OTU table) at the genus taxonomic level, generated by QIIME software. Dominant and rare OTUs in sequence libraries and in core taxa were defined at a threshold of 1% relative abundance (Gobet et al., 2010). Core taxa in sufu were visualized using the Pheatmap package in *R* (Kolde and Kolde, 2015). For phylogenetic analysis of the core taxa at higher taxonomic resolution than the genus level, representative sequences belonging to the OTUs of each core taxa (genus level) were extracted using QIIME and taxonomically classified after re-alignment with web-based SINA v1.2.11 (Quast et al., 2012). The top 10 closest relatives (minimum 95% pairwise similarity) for each sequence (OTU) were selected, aligned in web-based SINA v1.2.11, combined with the core taxa sequences, and imported into ARB (Ludwig et al., 2004). The average sequence divergence in core taxa assigned to a functional group and in each genus-level core taxon was calculated based on distance matrices in ARB.

### Statistical Analysis

Statistical analyses were conducted using SPSS 18.0 Software (SPSS Inc., Chicago, IL, United States). Statistical significance was estimated with two-way analysis of variance followed by the least significant difference test to determine the significance of differences between groups. For physicochemical characteristics, Student’s *t*-tests were applied to investigate the significance of differences in SPSS 18.0 Software (SPSS Inc., Chicago, IL, United States). Differences were considered to be significant when the *P* value was less than 0.05. Linear discriminant analysis (LDA) of effect size (LEfSe) was applied to determine the most discriminant taxa among two types of sufu samples (Segata et al., 2011), with a value for the statistical test equal to 0.05 and a logarithmic LDA score threshold of 4.0. In order to accurately predict and explain the relationships between bacterial data and chemical variables in sufu, aggregated boosted tree (ABT) analysis (with 5000 trees used for the boosting, 10-fold crossvalidation, and three-way interactions) was performed to quantitatively evaluate the relative influence of individual chemical factors on the bacterial community diversities using *R* package “gbm” (De’ath, 2007). The correlations between bacterial composition and physicochemical parameters, including metabolites and environmental factors, were analyzed using a heatmap.

### Sequence Accession

The bacterial 16S rRNA gene sequencing data are publicly available in the NCBI Short Read Archive under Bioproject accession number PRJNA601615.

## Results and Discussion

### Physicochemical Differences Between RS and WS

All sufu samples exhibited a pH range from 5.18 to 6.89 and salt concentration range from 6.92% to 13.31% (Table 1 and Supplementary Table S1). RS and WS samples contained AAN concentrations of 0.59% ± 0.18% and 1.00% ± 0.27%, respectively (Table 1). The bacterial abundances of the 16 sufu samples were enumerated by counting viable cells on PCA. The mean bacterial colony counts were approximately 2.4 × 10^6^ CFU/g for RS and 1.8 × 10^6^ CFU/g for WS (Table 1 and Supplementary Table S1), similar to a previous report (Han et al., 2001a). Differences were significant only for the content of AAN between RS and WS (Table 1).

**TABLE 1:** Chemical characteristics of sufu samples*.

Types	pH	NaCl (%)	Total acid (%)	Amino acid nitrogen (%)	Bacteria (log CFU/g)
RS	5.83 ± 0.53	8.07 ± 0.85	0.90 ± 0.27	0.59 ± 0.18 b	5.30 ± 1.44
WS	6.16 ± 0.74	9.17 ± 1.97	0.89 ± 0.36	1.00 ± 0.27 a	4.91 ± 1.08

*Chemical characteristics are represented as mean ± SD obtained across triplicate measurements. Means with different superscript letters are significantly different horizontally (*P* < 0.05).

**FIGURE 1:**
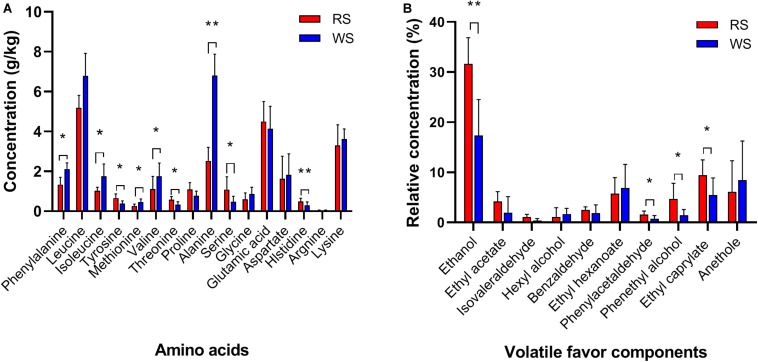
Profiles of amino acids **(A)** and volatile flavor compounds **(B)** in sufu samples. Averages ± SEMs of samples in each group are expressed in each column. The significance cutoff for the corrected *P* values determined using the Benjamini-Hochberg FDR procedure was set at 0.05. * *P* < 0.05, ** *P* < 0.01 for the indicated comparisons.

Next, we evaluated differences in metabolites, including organic acids, FAAs, and VFCs, using chromatography and MS. Lactic acid was the most abundant organic acid, accounting for approximately 1.969 ± 0.300 g/kg in RS and 2.790 ± 0.612 g/kg in WS, followed by acetic acid (average contents of 0.715 ± 0.102 g/kg in RS and 0.855 ± 0.090 g/kg in WS) and citric acid (0.805 ± 0.050 g/kg in RS and 0.679 ± 0.153 g/kg in WS; Supplementary Figure S2). Succinic acid and propionic acid were not detectable in all sufu samples. Lactic acid, acetic acid, and citric acid have previously been reported as the predominated organic acids in sufu (Moy and Chou, 2010). However, there were no significant differences in these high-abundance organic acids between RS and WS in our study. In contrast, less abundant organic acids (e.g., pyruvic acid and malic acid, with contents less than 0.200 g/kg) showed significant differences (*P* < 0.05) between RS and WS. Moreover, in FAA profiles, leucine (average contents of 5.19 ± 0.62 g/kg in RS and 6.79 ± 1.13 g/kg in WS), alanine (2.53 ± 0.67 g/kg in RS and 6.80 ± 1.08 g/kg in WS), glutamic acid (4.50 ± 1.00 g/kg in RS and 4.14 ± 1.12 g/kg in WS), and lysine (3.30 ± 1.04 g/kg in RS and 3.62 ± 0.51 g/kg in WS) were the predominant amino acid species in RS and WS. The levels of phenylalanine, isoleucine, tyrosine, methionine, valine, threonine, alanine, serine, and histidine differed significantly (*P* < 0.05) between the two types of sufu (Figure 1A). The total amount of FAAs in WS (average 32.43 ± 3.28 g/kg) was higher than that in RS (average 25.48 ± 2.59 g/kg), consistent with a previous study in which FAA levels were found to be higher in WS (89.5 g/kg dry matter) than in RS (64.4 g/kg dry matter; Han et al., 2004b).

In total, 147 VFCs were identified in RS and WS; these VFCs included 44 esters, 25 alcohols, 13 aldehydes, 14 ketones, two acids, seven phenols, and 42 other unclassified compounds (Supplementary Table S2). The dominant VFCs (with relative concentrations higher than 1%) were ethanol, ethyl acetic acid, isovaleraldehyde, hexyl alcohol, ethyl hexanoate, phenylacetaldehyde, phenylethyl alcohol, ethyl caprylate, and anethole (Figure 1B); ethanol, phenylacetaldehyde, phenethyl alcohol, and ethyl caprylate showed significant differences between RS and WS (*P* < 0.05; Figure 1B). The sufu were the commercial products collected from markets, that absent of the constitute and concentration of dressing mixture added in the ripening stage of fermentation. The ingredients of dressing mixture vary with social customs, climate, locations and so on. The most common dressing mixture used consists of angkak, alcoholic beverage, salt, sugar, flour (bean paste), and spices (Han et al., 2001b). To supply a special flavor for sufu, kinds of dressing mixtures can be added into the production of sufu, which may impact the VFCs concentration in sufu. However, the influence of dressing mixture on VFCs should be further examined, and also the effect on the microbial community structure should be explored in detail in the future. The highest proportion of ethanol (average 24.49% ± 3.71%) may have resulted from the addition of a dressing mixture during the ripening stage of sufu because this dressing mixture contained large amounts of alcoholic beverage and exhibited high microbial metabolism (Hwan and Chou, 1999; Han et al., 2003). The ethanol in sufu can be originated from the dressing mixture or generated by the ethanol-producing microorganisms. Because sufu samples were purchased from markets, the information of the added dressing mixture was missing in this study. Our results showed that ethanol was the most abundant VFCs in sufu, and its concentration was significantly different between red and WS (Figure 1B). The similarity of the production process of red and WS is convinced, and WS has similar ingredients as RS in the dressing mixture but without angkak (Han et al., 2001b). The sufu is a typical mould-fermented sufu in China. It is manufactured by first cultivating a fungus such as Actinomucor, Mucor, or Rhizopus on the surface of tofu cubes to prepare the pehtze. In a study of characterizing dynamic changes of the fungal and bacterial communities during the production of RS, Cryptococcus and Actinomucor were the most abundant genera at salt-pehtze stage. But after pouring dressing mixture, Monascus and Aspergillus became the main genera during the ripening fermentation stage (Xu et al., 2020). Although Pichia (ethanol-producing yeast) was also the main genera (∼5%) in the stage of ripening (Xu et al., 2020), the main chemical compounds such as ethanol, ethylene glycol, glucose, isopropanol, and mannitol were stable during the ripening fermentation stage (Liu et al., 2018). Therefore, the majority of ethanol in sufu should not be generated by yeast. Based on these results, we speculate the significant difference of ethanol concentration between red and WS came from the of the dressing mixture. In addition to ethanol, ester compounds (e.g., ethyl acetic acid, ethyl hexanoate, and ethyl caprylate) were also predominant in sufu, similar to the results of previous studies (Chung et al., 2005; Xie et al., 2018; Liang et al., 2019). These compounds are characterized by fruit-like and floral aromas, which are formed mainly by the esterification of alcohols and organic acids (Erten et al., 2007), and by the contribution of microbial activity during the fermentation and storage period (Wang et al., 2015b).

### Differences in Bacterial Communities Between RS and WS

To determine differences among bacterial communities of the two types of sufu, we analyzed the sequences of bacterial 16S rRNA gene amplicons using high-throughput sequencing technology. After quality control, denoising, and chimera removal, a dataset consisting of 1,540,511 filtered high-quality 16S rRNA gene sequences was generated, and an average of 96,282 sequences was obtained for each individual sample (range: 55,230–145,310; Supplementary Table S3). All sequences were clustered into OTUs at 97% sequence similarity. The number of OTUs per sample ranged from 74 to 188 (Supplementary Table S3). Bacterial 16S rRNA gene sequence numbers were normalized to 55,230 reads before describing the alpha diversity and composition characteristics. Goods’ coverage values, indicating estimates of sampling completeness, were 0.9999095–1.0000000 at a 97% similarity level, suggesting that the sequencing reads obtained in each sample were sufficient to analyze bacterial diversity. The microbial alpha diversity, as measured by Chao1, Shannon, and Simpson indexes, was estimated using the QIIME2 platform. Briefly, the Chao1 indexes were 131.37 ± 39.08 and 152.18 ± 28.33 for RS and WS samples, respectively; the observed OTUs were 130.63 ± 38.41 and 151.50 ± 27.6 for RS and WS samples, respectively; and the PD indices were 6.45 ± 1.09 and 7.31 ± 1.31 for RS and WS samples, respectively. Shannon and Simpson diversity indices were significantly higher in WS than in RS (*P* = 0.046 and 0.016, respectively; Supplementary Table S3). However, the number of observed OTUs and the values of Chao1, ACE, and PD indexes did not differ significantly between RS and WS samples (Supplementary Table S3).

**FIGURE 2:**
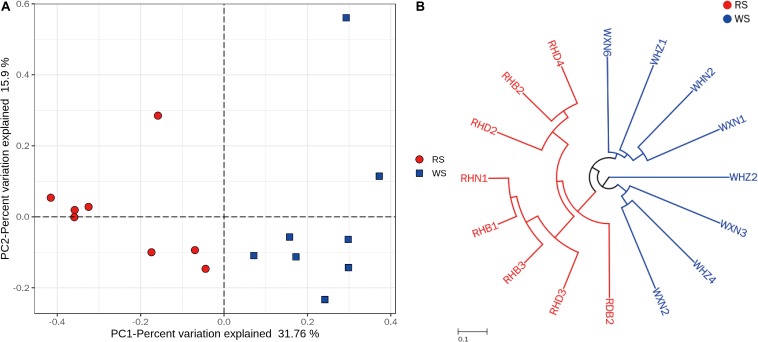
Clustering of the bacterial communities in sufu samples. **(A)** PCoA of bacterial communities in red (RS) and white (WS) sufu samples, based on Bray–Curtis distances. The percent variation of the plotted principal component is indicated on the axes. **(B)** UMPGA hierarchical cluster analysis dendrograms based on OTUs of the 16 commercial sufu samples. All 16S rRNA samples were subsampled to 55,230 sequences before Bray–Curtis distance calculation.

Principal coordinate analysis and UPGMA hierarchical clustering were calculated after subsampling (55,230 sequences) in order to assesses differences in microbial community structures between RS and WS samples. PCoA and UPGMA revealed that RS samples grouped together in one cluster, whereas WS samples grouped in another cluster, indicating that the bacterial communities were significantly different between RS and WS (Figure 2). These findings could be related to the addition of red colorant (angkak) during the process of RS fermentation (Ho et al., 1989; Han et al., 2001a). Angkak contains antibacterial substances (such as citrinin and angkalactone; Blanc et al., 1995; Jùzlová et al., 1996), which may strongly shape the bacterial community structure, particularly for LAB (Han et al., 2001a). Indeed, previous reports have shown that citrinin from angkak inhibits the growth of *Streptococcus*, *Bacillus*, and *Pseudomonas* (Wong and Bau, 1977). Compared with WS, RS contained much higher amounts of alcohol, esters, and acids (Figure 1B), which may also influence the bacterial communities in sufu.

**FIGURE 3:**
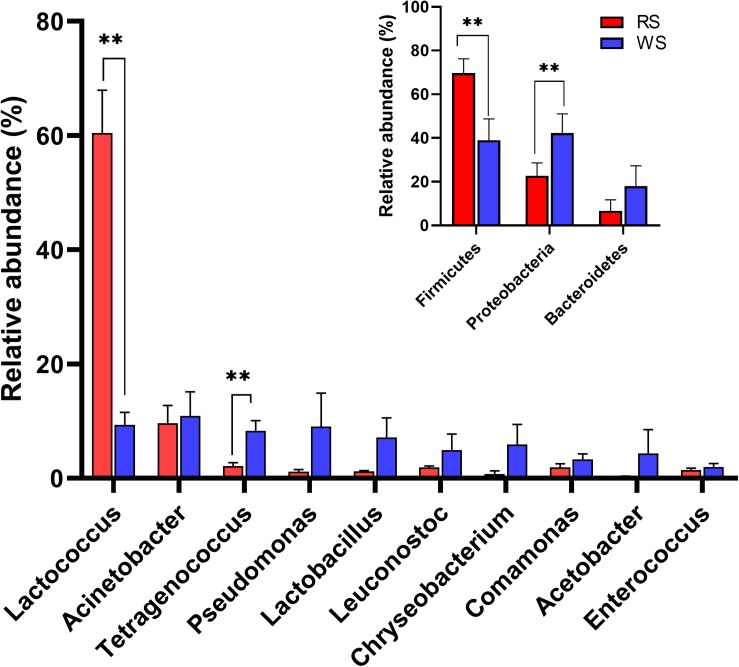
Taxonomic composition of bacterial communities showing differences between red sufu (RS) and white sufu (WS). The data revealed phylum (inset figure) and genus (main figure) level classifications for 16S rRNA gene sequences. 16S rRNA gene sequences longer than 250 bp were classified using the RDP naïve Bayesian rRNA Classifier at an 80% confidence threshold. Averages ± SEMs of samples in each group are expressed in each column. The significance cutoff for the corrected *P* value determined using the Benjamini-Hochberg FDR procedure was set at 0.01. ** *P* < 0.01 for the indicated comparisons.

To investigate the bacterial community composition of sufu samples, bacterial 16S rRNA gene sequences were classified at both the phylum and genus levels (Figure 3 and Supplementary Figures S3, S4). At the phylum level, the relative abundances of three phyla were higher than 1%. Specifically, Firmicutes (average ± standard error of the mean [SEM]: 69.71% ± 6.51%), Proteobacteria (22.72% ± 5.89%), and Bacteroidetes (6.60% ± 5.13%) were the predominant phyla in RS samples, accounting for 99.03% of the total microbiota. In contrast, in WS samples, the abundances of Proteobacteria (42.18% ± 8.84%), Firmicutes (38.94% ± 9.81%), and Bacteroidetes (17.99% ± 9.30%) differed (Figure 3). RS and WS samples also exhibited different genus-level profiles, with *Lactococcus* (60.43% ± 7.53%), *Acinetobacter* (9.62% ± 3.11%) and *Tetragenococcus* (2.16% ± 0.57%) representing the most abundant genera in RS samples (Figure 3). In contrast, *Acinetobacter* (10.92% ± 4.24%), *Lactococcus* (9.31% ± 2.20%), *Pseudomonas* (9.04% ± 5.88%), *Tetragenococcus* (8.31% ± 1.78%), *Lactobacillus* (7.08% ± 3.49%), and *Chryseobacterium* (5.89% ± 3.54%) were the predominant genera in WS samples (Figure 3). Previous reports have shown that *Lactococcus*, *Acinetobacter*, *Tetragenococcus*, *Pseudomonas*, and *Lactobacillus* are the dominant bacteria and crucial contributors during the production of fermented foods, such as cheese, sufu, soy sauce, liquor, tea, and vinegar (Tanaka et al., 2012; Wang et al., 2016; Gu et al., 2018; Huang et al., 2018; Li et al., 2019; Liang et al., 2019; Liu and Qiao, 2019). At the phylum level, the relative abundances of *Firmicutes* and *Proteobacteria* were significantly different between these two types of sufu samples (*P* < 0.01; Figure 3). Moreover, *Lactococcus* and *Tetragenococcus* showed significantly different abundances between RS and WS samples (*P* < 0.01; Figure 3).

Next, biomarker analysis using the LEfSe method was performed to determine the classified bacterial taxa with significantly different abundances between RS and WS. As shown in Supplementary Figure S5, six bacterial clades showed statistically significant differences, with an LDA score of 4.0. Specifically, *Lactococcus* (genus), *Streptococcaceae* (family), and *Lactobacillales* (order) were abundant in RS samples. In contrast, *Enterococcaceae* (family), *Tetragenococcus* (genus), and *Bacillales* (order) were common in WS samples. The significantly different abundances of *Lactococcus* and *Tetragenococcus* in RS and WS could explant the divergence of chemical characteristics between the two types of sufu. Our results also showed that *Lactococcus* was the dominant microorganism. *Lactococcus* mainly affects the conversion of amino acids to flavor compounds in cheese (Yvon et al., 1997; Kieronczyk et al., 2006). *Tetragenococcus* are halophilic LAB that occur mainly in fermented foods, such as doubanjiang-meju (Li et al., 2017), doenjang (Kim et al., 2009), and sufu (Xie et al., 2018; Liu and Qiao, 2019). Importantly, *Tetragenococcus halophilus* is essential for the production of desirable volatile compounds during soy sauce production (Devanthi et al., 2018).

**FIGURE 4:**
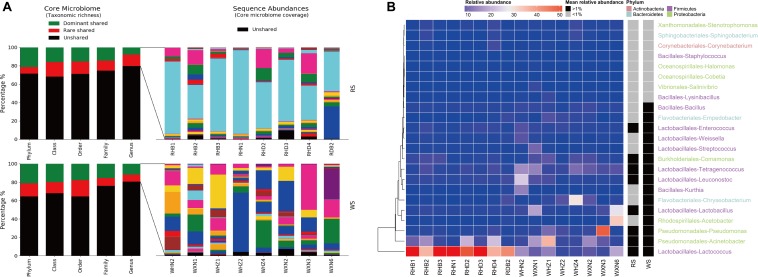
Richness, abundance, and identity of bacterial taxa shared between RS and WS samples. **(A)** Richness and sequence coverage of shared taxa in RS and WS. **(B)** Shared genera and their absolute abundances. Sequence abundances were log-transformed and colored from red to black to present higher-to-lower abundances. Heatmap boxes without any sequences were assigned 10^– 6^ (darkest green).

Shared taxa (at the genus level) present in all sufu samples at more or less than 1% relative abundance were defined as dominant or rare core taxa, respectively (Figure 4). At the genus level, RS comprised 29 core genera in four phyla, accounting for 20.28% ± 3.30% of the total sequence abundance (Figure 4A). In the WS bacterial communities, 28 core taxa were identified, comprising 19.58% ± 2.11% of the total sequence abundance (Figure 4A). All RS dominant core taxa (representing 79.73% ± 15.01% of the shared sequence abundances) were also in the WS core, and 15 taxa constituted the dominant core of WS microbial communities (73.35% ± 24.14% of the shared sequence abundance; Figure 4B). Seven of the rare core taxa in the RS were dominant core taxa in the WS, including *Bacillus*, *Empedobacter*, *Weissella*, *Streptococcus*, *Kurthia*, *Chryseobacteriuma*, and *Acetobacter*. For RS and WS communities, dominant cores were dominated by *Enterococcus*, *Tetragenococcus*, *Leuconostoc*, *Lactobacillus*, *Pseudomonas*, *Acinetobacter*, and *Lactococcus* (Figure 4B). Notably, these bacteria were also found to be dominant in other reports of sufu and other food fermentation processes (Kang et al., 2003; Feng et al., 2013; Li et al., 2017; Gu et al., 2018; Huang et al., 2018; Xie et al., 2018).

### Correlations Between Bacterial Communities and Metabolites

**FIGURE 5:**
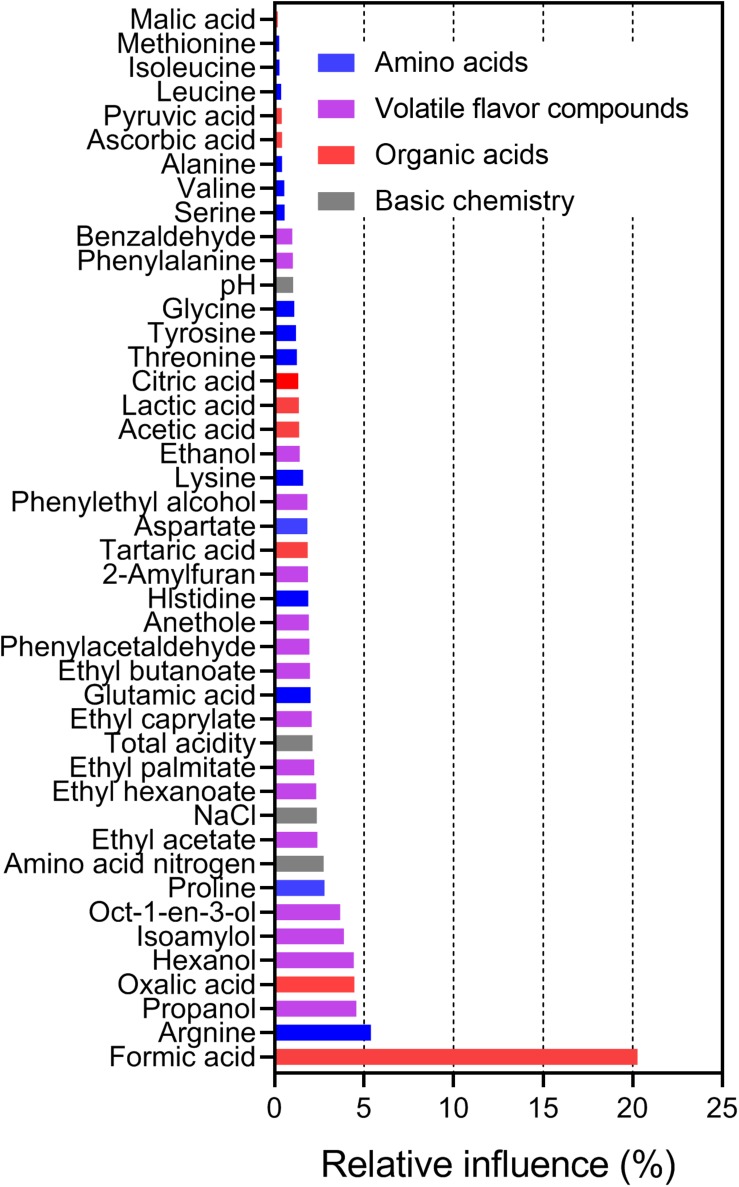
Relative influence (%) of chemical variables on the Chao1 index as evaluated by the ABT analysis, with 5000 trees used for the boosting, 10-fold cross-validation, and three-way interactions.

Next, the relationships between species richness and metabolites were analyzed by ABT analysis (De’ath, 2007). The results showed that formic acid had the highest relative influence (20.27%) on the Chao1 index of bacterial communities, following by arginine (5.38%), propanol (4.57%), oxalic acid (4.46%), and hexanol (4.43%; Figure 5). These findings implied that formic acid may be a key factor driving the species richness in sufu. Indeed, acidity is an important factor in microbial population succession (Wang et al., 2015a), and formic acid is key factor for bacterial growth and community formation in cheese (Oh et al., 2016). In a study of mixed silage of air-dried corn stover and cabbage waste, formic acid was found to have negative effects on the OTU number of 16S rRNA genes and to increase the relative abundance of *Firmicutes* in silage (Ren et al., 2018). In our study, *Lactobacillus* was the predominant genus in WS (average ± SEM: 9.87% ± 3.49%) and RS (0.36% ± 0.13%) samples in this study (Figure 3). Moreover, formic acid can stimulate RNA synthesis in cells (Suzuki et al., 1986), which can also influence the bacterial community structure in sufu. Evaluation of the relative influence of the bacterial composition on the concentration of formic acid showed that *Streptococcaceae* and *Moraxellaceae* had the highest relative influence (12.84% and 8.75%, respectively; Supplementary Figure S6). *Streptococcaceae*, mainly represented by *Lactococcus* and *Streptococcus*, is highly correlated with formic acid in the fermentation of probiotic cheeses (Oh et al., 2016). Additionally, *Moraxellaceae*, mainly represented by *Acinetobacter*, is also abundant in other fermented foods (Wang et al., 2015b; Li et al., 2017; Huang et al., 2018). Previous studies have indicated that the genus *Acinetobacter* is well known for its capacity to secrete esterolytic enzymes (Ahmed et al., 2010), and is positively related to flavor compounds, particularly esters (Huang et al., 2018). However, relatively little is known regarding the roles of *Streptococcaceae* and *Moraxellaceae* in formic acid generation in sufu, and the effects and mechanisms of formic acid on sufu bacterial communities remain unclear.

**FIGURE 6:**
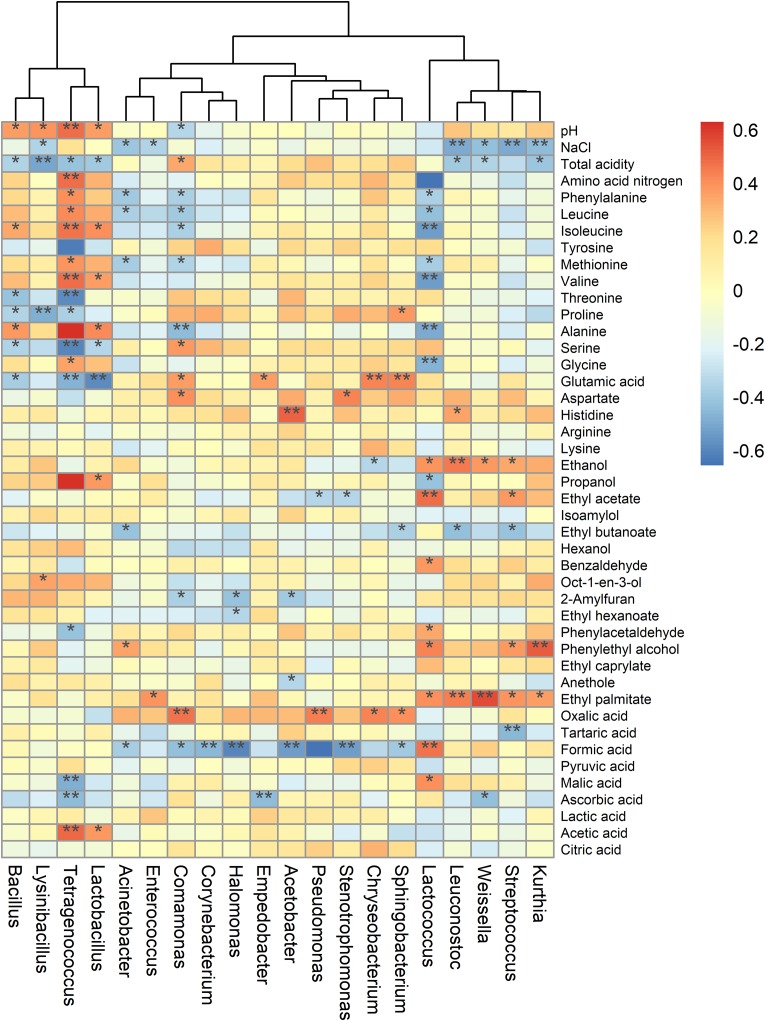
Heatmap of the correlations of bacterial genera with metabolites and other physicochemical parameters. Strength (Spearman’s ρ value) and significance of correlations were showed as color in shades (red, positive correlation; blue, and negative correlation). The heatmap values ranged from + 0.6 to -0.6. The values above/below zero represent positive/negative correlations between bacterial genera and the parameters analyzed. * *P* < 0.05, ** *P* < 0.01 for the indicated comparisons.

To elucidate the correlations between bacterial communities and metabolites, Pearson’s correlation coefficients of the relative abundances of bacterial genera and the contents of metabolites were calculated (Figure 6). The results showed that *Lactococcus* were positively correlated with VFCs (ethanol, ethyl acetate, and benzaldehyde, etc.) and organic acid (formic acid and malic acid), but negatively related to amino acid (isoleucine, alanine, valine, and glycine, etc.) (all *P* < 0.05). Previous studies have shown that *Lactococcus* is positively correlated with flavor compounds, particularly acids and esters (Huang et al., 2018; Xie et al., 2018). It was also reported that *Lactococcus lactis* IO-1grew well with xylose as the carbon source, and produced formic acid (Tanaka et al., 2002). Besides, the abundances of *Tetragenococcus* and *Comamonas* were significantly related to the species of amino acids (Figure 6), indicated their important roles in the production and transformation of amino acids in sufu fermentation. *Tetragenococcus* occurred dominantly in fermented foods, such as, doenjang, sufu and soy sauce, which is essential for the production of desirable volatile compounds (Kim et al., 2009; Devanthi et al., 2018; Xie et al., 2018). It was also demonstrated that *Comamonas testosteroni* can produce alanine from aspartate by aspartate-β-decarboxylase (Nakamori, 2017). Additionally, we found that *Leuconostoc* had a mostly positive relationship with ethanol, suggested it can tolerate elevated levels of ethanol. Besides, it has been proven that *Leuconostoc mesenteroides* could convert acetaldehyde to ethanol and acetate (Liu et al., 1997). However, the results obtained in this study were based on commercial sufu. Therefore, further studies should be conducted to identify important species and their interactions in flavor generation during sufu fermentation with different materials, starters, or technologies.

## Conclusion

In this study, we examined the bacterial communities and metabolites of different types of sufu by coupling high-throughput 16S rRNA gene amplicon target sequencing and metabolomic approaches. The correlations between bacterial community and metabolites were also analyzed in sufu. To the best of our knowledge, this is the first report to comprehensively evaluate the molecular ecology and correlations between microbiota and metabolites in RS and WS. Our work provides in-depth insights into the roles of bacterial communities in the generation of metabolites in sufu. Further studies are needed to confirm our findings with regard to microbial communities, flavor components, enzymes, and their correlations using a meta-omics approach.

## Data Availability Statement

All datasets generated for this study are included in the article/Supplementary Material.

## Author Contributions

GT, MH, and MY conceived and designed the experiments. XL and ZP performed most of the experiments. ML, LL, and MY supervised the execution of the experiments. GT and MH wrote the manuscript. All authors read and approved the final manuscript.

## Conflict of Interest

MY was employed by Zhuhai Da Hengqin Science and Technology Development Co., Ltd. The remaining authors declare that the research was conducted in the absence of any commercial or financial relationships that could be construed as a potential conflict of interest.

## Footnotes

1 http://www.novogene.com

## Supplementary Material

FIGURE S1 The workflow diagram of sufu production.

FIGURE S2 Profiles of organic acids in sufu samples. Averages ± standard deviations for samples in each group are expressed in each column. The significance cutoff for the corrected *P* value using the Benjamini-Hochberg FDR procedure was set at 0.05. **P* < 0.05 for the indicated comparisons.

FIGURE S3 Relative abundance percentages of bacterial phyla among different sufu samples. The relative abundance of each taxon was defined as the percentage of the same taxon to the corresponding total sequences for each sample. Some classes with abundances of less than 1% were summarized as “other.”

FIGURE S4 Relative abundance percentages of bacterial genera among different sufu samples. The relative abundance of each taxon was defined as the percentage of the same taxon to the corresponding total sequences for each sample. Some classes with abundances of less than 1% were summarized as “other.”

FIGURE S5 The microbial communities of red (RS) and white (WS) sufu samples were analyzed using the LDA effect size algorithm to determine the optimal characteristic taxa and rank them according to the effect size. LDA scores identified the size of differentiation; the score threshold was 4.0.

FIGURE S6 Relative influence of the bacterial composition on formic acid.

TABLE S1 Characteristics of the physicochemical parameters of sufu.

TABLE S2 Characteristics of volatile favor compounds (VFCs) in RS and WS.

TABLE S3 Statistical analysis of bacterial 16S rRNA sequencing data for the sufu samples.
