# Supplementary figures and images for: High-Throughput Sequencing and Metabolomics Reveal Differences in Bacterial Diversity and Metabolites Between Red and White Sufu
Source: Front Microbiol. 2020 Apr 22;11:758. doi: 10.3389/fmicb.2020.00758 (PMC7188790; doi:10.3389/fmicb.2020.00758)

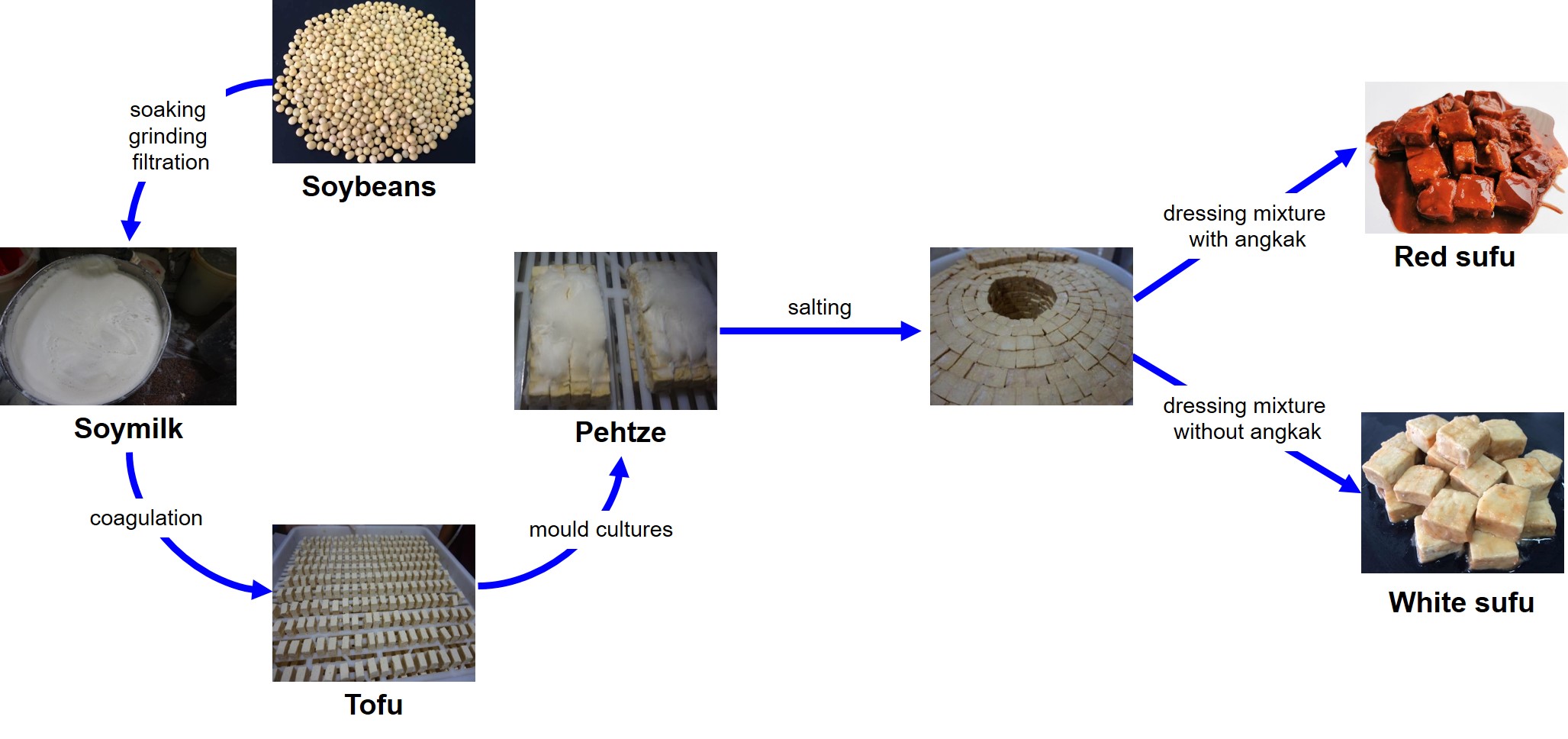

Supplement: FIGURE S1 — The workflow diagram of sufu production. [file Image_1.JPEG]

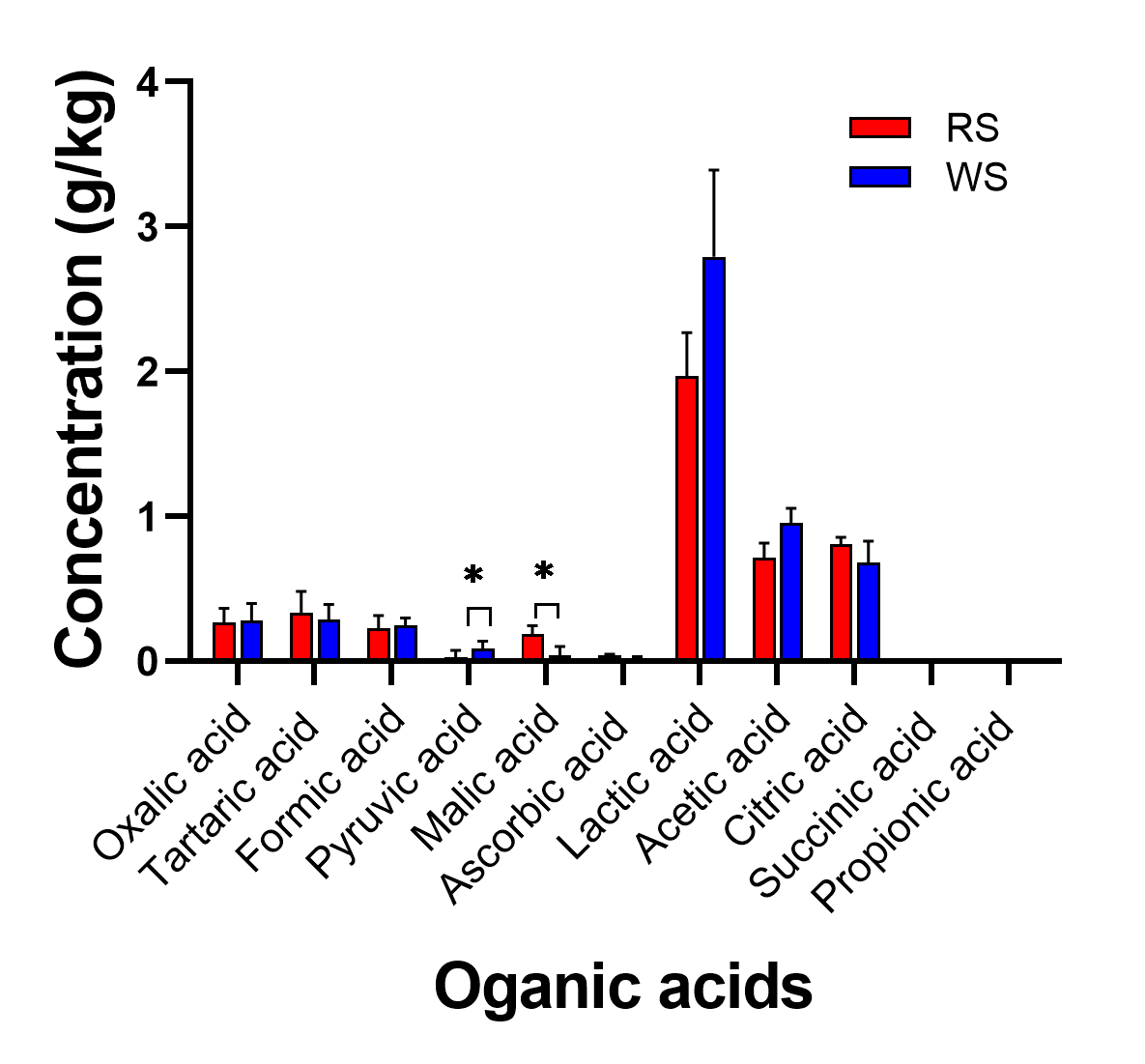

Supplement: FIGURE S2 — Profiles of organic acids in sufu samples. Averages ± standard deviations for samples in each group are expressed in each column. The significance cutoff for the corrected P value using the Benjamini-Hochberg FDR procedure was set at 0.05. *P < 0.05 for the indicated comparisons. [file Image_2.TIF]

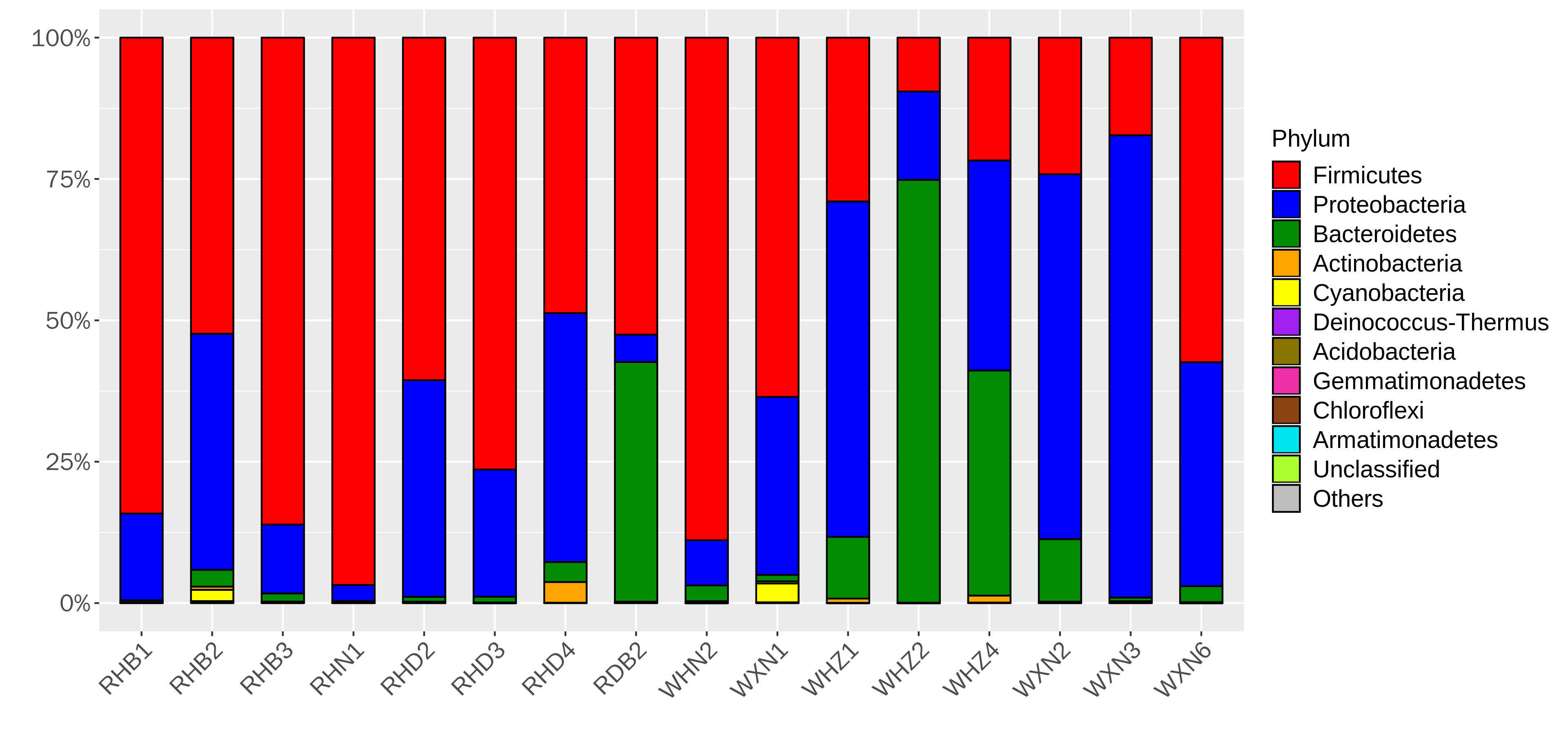

Supplement: FIGURE S3 — Relative abundance percentages of bacterial phyla among different sufu samples. The relative abundance of each taxon was defined as the percentage of the same taxon to the corresponding total sequences for each sample. Some classes with abundances of less than 1% were summarized as “other.” [file Image_3.TIF]

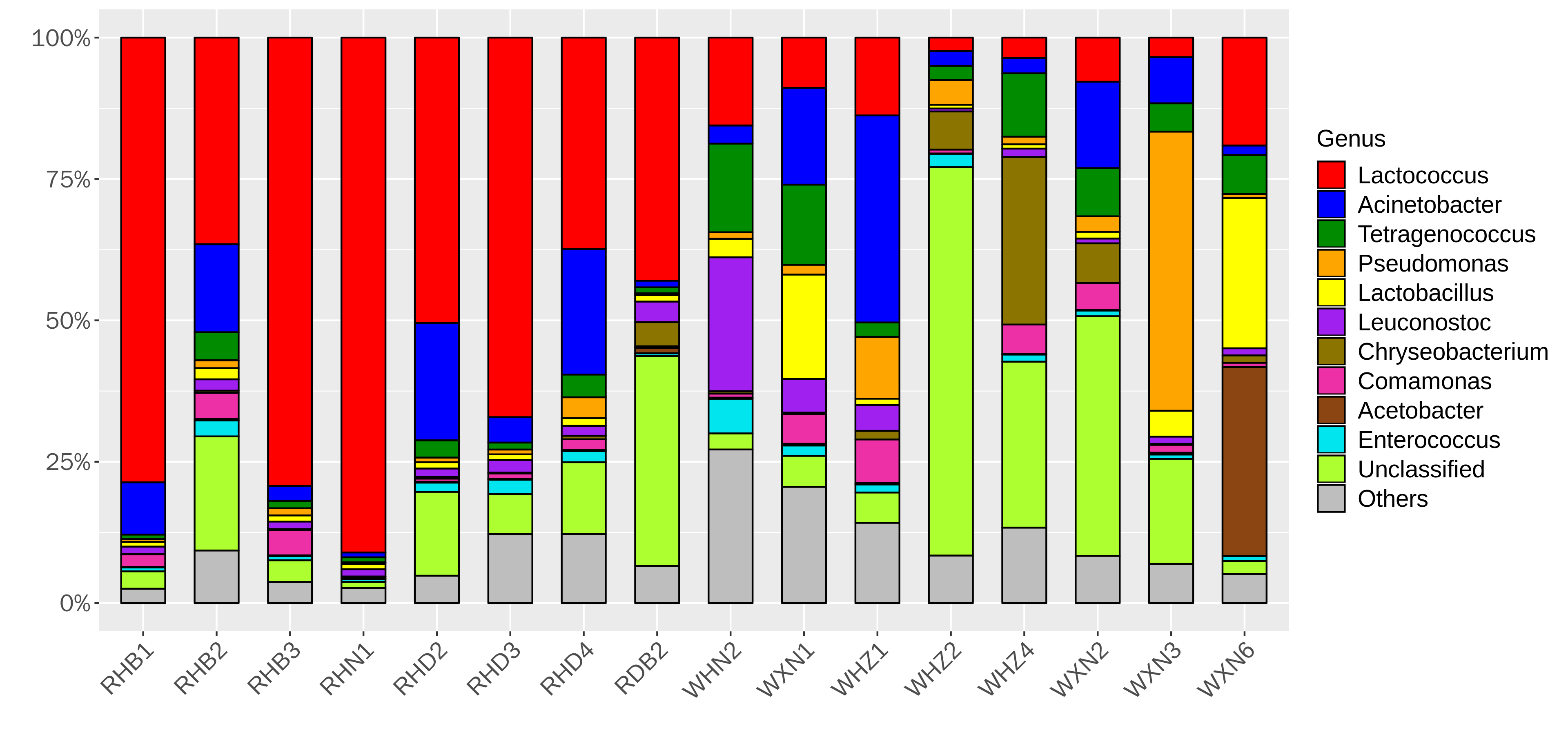

Supplement: FIGURE S4 — Relative abundance percentages of bacterial genera among different sufu samples. The relative abundance of each taxon was defined as the percentage of the same taxon to the corresponding total sequences for each sample. Some classes with abundances of less than 1% were summarized as “other.” [file Image_4.TIF]

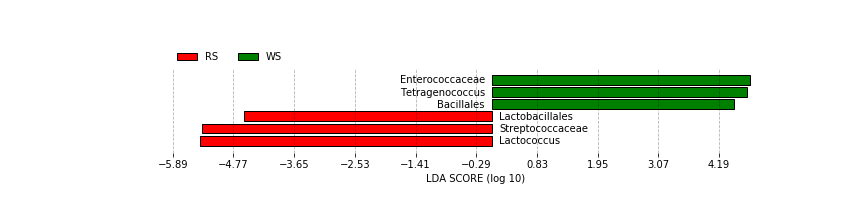

Supplement: FIGURE S5 — The microbial communities of red (RS) and white (WS) sufu samples were analyzed using the LDA effect size algorithm to determine the optimal characteristic taxa and rank them according to the effect size. LDA scores identified the size of differentiation; the score threshold was 4.0. [file Image_5.TIF]

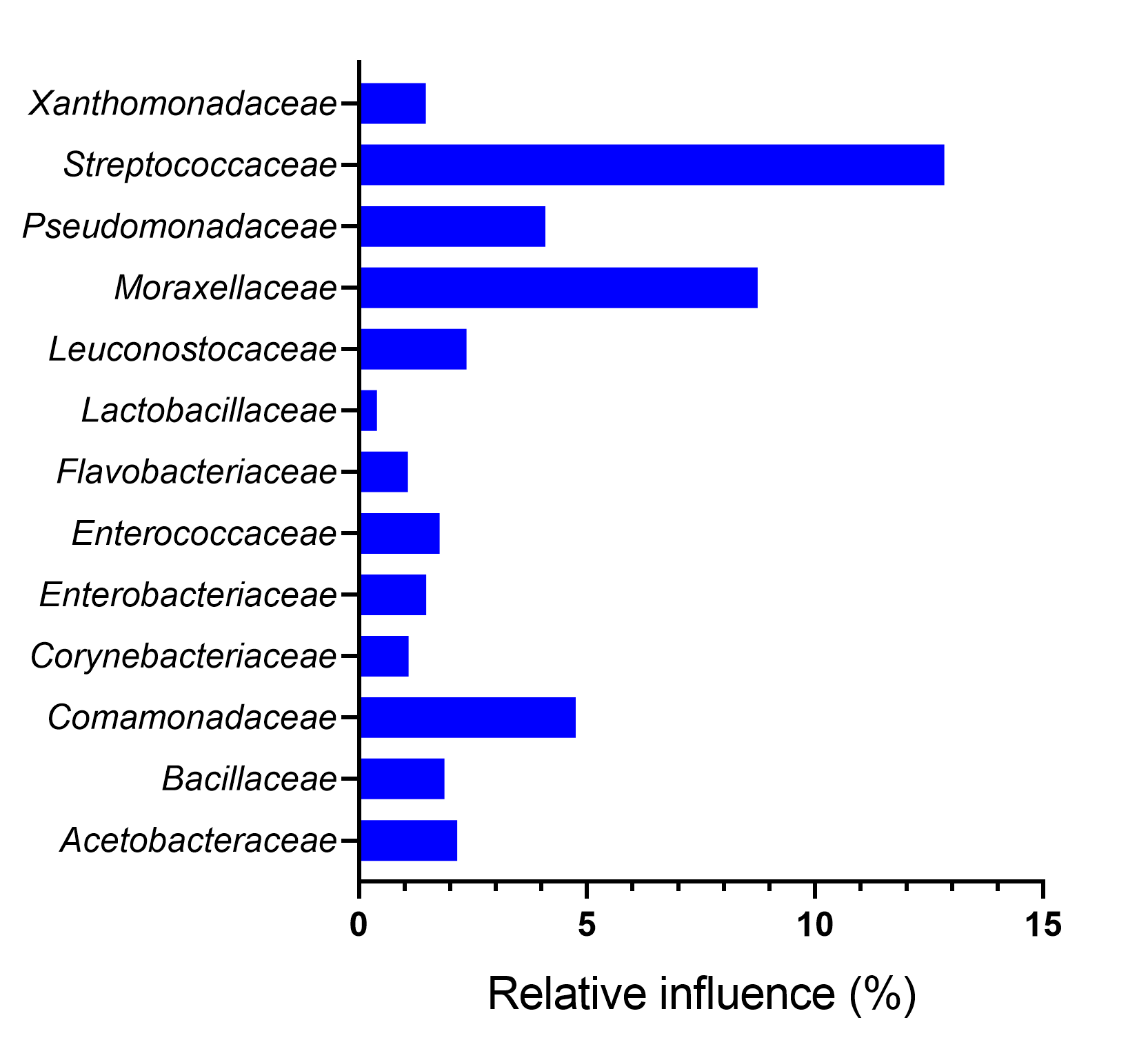

Supplement: FIGURE S6 — Relative influence of the bacterial composition on formic acid. [file Image_6.TIF]
